# SGLT2i improves kidney senescence by down‐regulating the expression of LTBP2 in SAMP8 mice

**DOI:** 10.1111/jcmm.18176

**Published:** 2024-03-07

**Authors:** Lu Zeng, Jie Li, Fanfan Gao, Yangyang Song, Limin Wei, Ning Qu, Shengnan Chen, Xue Zhao, Zitong Lei, Wenya Cao, Lei Chen, Hongli Jiang

**Affiliations:** ^1^ Department of Critical Care Nephrology and Blood Purification The First Affiliated Hospital of Xi'an Jiaotong University Xi'an Shannxi China

**Keywords:** dapagliflozin, kidney, LTBP2, SAMP8, senescence, SGLT2i

## Abstract

Senescent kidney can lead to the maladaptive repairment and predispose age‐related kidney diseases. Here, we explore the renal anti‐senescence effect of a known kind of drug, sodium‐dependent glucose transporters 2 inhibitor (SGLT2i). After 4 months intragastrically administration with dapagliflozin on senescence‐accelerated mouse prone 8 (SAMP8) strain mice, the physiologically effects (lowering urine protein, enhancing glomerular blood perfusion, inhibiting expression of senescence‐related biomarkers) and structural changes (improving kidney atrophy, alleviating fibrosis, decreasing glomerular mesangial proliferation) indicate the potential value of delaying kidney senescence of SGLT2i. Senescent human proximal tubular epithelial (HK‐2) cells induced by H_2_O_2_ also exhibit lower senescent markers after dapagliflozin treatment. Further mechanism exploration suggests LTBP2 have the great possibility to be the target for SGLT2i to exert its renal anti‐senescence role. Dapagliflozin down‐regulate the LTBP2 expression in kidney tissues and HK‐2 cells with senescent phenotypes. Immunofluorescence staining show SGLT2 and LTBP2 exist colocalization, and protein‐docking analysis implies there is salt‐bridge formation between them; these all indicate the possibility of weak‐interaction between the two proteins. Apart from reducing LTBP2 expression in intracellular area induced by H_2_O_2_, dapagliflozin also decrease the concentration of LTBP2 in cell culture medium. Together, these results reveal dapagliflozin can delay natural kidney senescence in non‐diabetes environment; the mechanism may be through regulating the role of LTBP2.

## 1 INTRODUCTION

Deteriorating kidney function can be more frequently occurred among elderly population.^1^ Ageing kidney is associated with susceptibility to acute kidney failure (AKI) and easy to progress into chronic kidney disease (CKD).^2^, ^3^ Repurposing drug having potential for delaying kidney senescence and improving outcome of kidney diseases will be beneficial for maximizing the value of the drug. Sodium‐dependent glucose transporters 2 inhibitor (SGLT2is) are new category drug for lowering blood glucose though increasing urinary glucose excretion.^4^ Several studies have testified that SGLT2i might prolong mice lifespan^5^, ^6^; the evidence about direct beneficial role of SGLT2i on kidney natural senescence still be scarce.

In current study, we hypothesized SGLT2i could delay natural kidney senescence under normal blood glucose. Senescence‐accelerated mouse prone 8 (SAMP8) strain mice were selected as kidney natural senescence mouse model, administrated with dapagliflozin for 4 months to demonstrate whether the process of kidney natural senescence can be delayed by dapagliflozin. LTBP2 (Latent transforming growth factor beta binding protein 2) belongs to the family of latent transforming growth factor (TGF)‐beta binding proteins (LTBP), which are extracellular matrix proteins with multi‐domain structure.^7^ In current study, we observed the expression of LTBP2 was down‐regulated during the renal anti‐senescence process exerted by dapagliflozin, further functional and mechanistical research results revealed that LTBP2 might be the target gene of SGLT2i for playing its renal anti‐senescence role.

## 2 RESULTS

### 2.1 SAMP8 mice exhibit kidney senescence features

**FIGURE 1:**
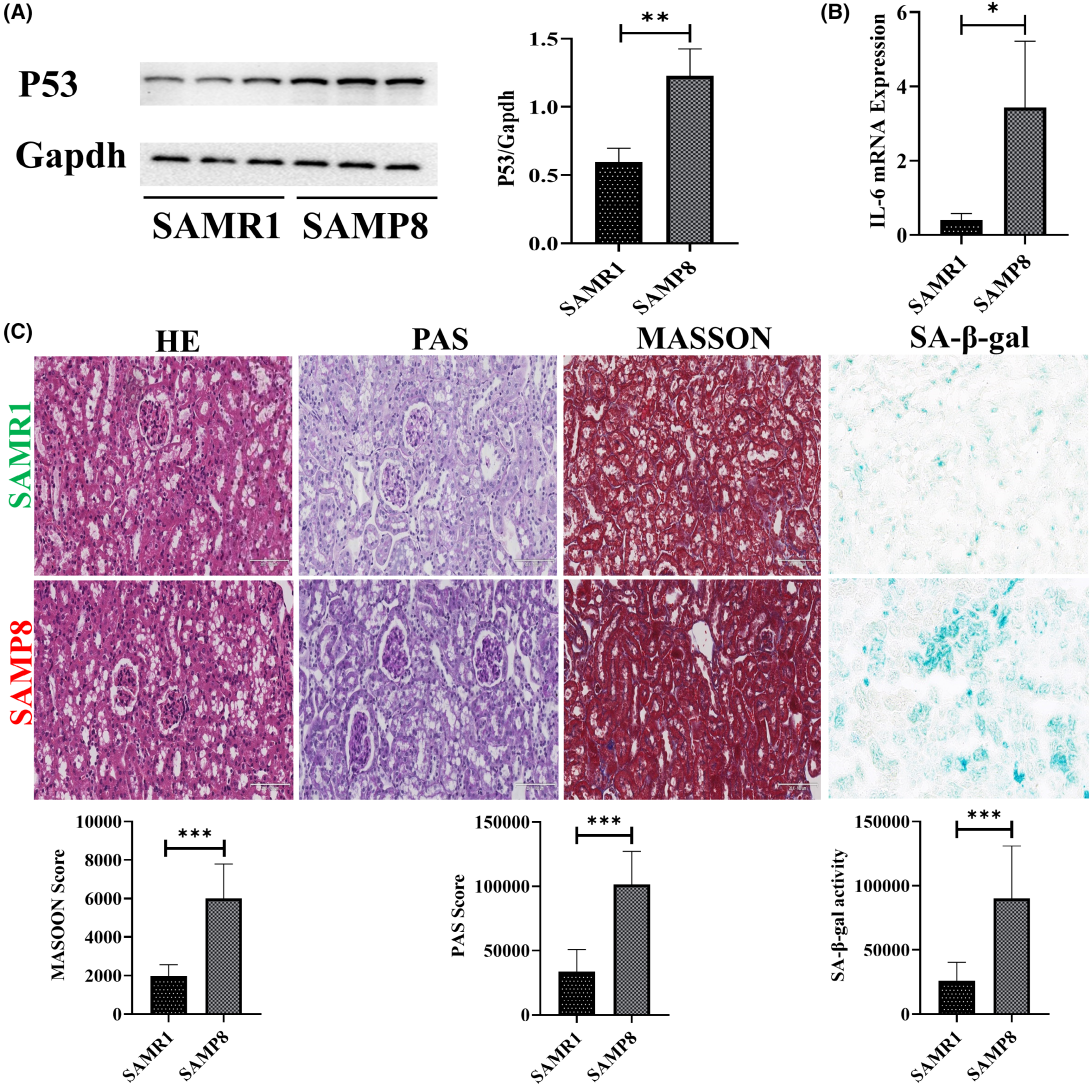
SAMP8 mice exhibited features of kidney senescence. (A) Representative western blotting images and quantification result of P53. (B) IL‐6 mRNA expression. (C) Representative kidney sections stained with haematoxylin–eosin (HE, scale bar 100 μm), periodic acid‐Schiff (PAS, scale bar 100 μm), Masson trichrome (MASSON, scale bar 100 μm), senescence‐associated β‐galactosidase (SA‐β‐gal, scale bar 200 μm) and quantification results of PAS, MASSON and SA‐β‐gal activity. *p < 0.05, **p < 0.01 and ***p < 0.001.

Seven‐month‐old SAMR1 and SAMP8 mice kidneys were separately sampled for testing the kidney senescence characters. Expression of P53 increased in SAMP8 compared with SAMR1 as measured by western blotting (Figure 1A). IL‐6 mRNA expression was also elevated in SAMP8 (Figure 1B). Pathological and histological examination indicated SAMP8 mice kidney sections exhibited much more disordered and dilated tubules, more severe glomerulosclerosis and more interstitial fibrosis. SA‐β‐gal staining also exhibited higher activity in SAMP8 kidney tissue (Figure 1C). These results all demonstrated that SAMP8 mouse model exhibited some features of kidney senescence during the natural kidney ageing process compared with SAMR1 mouse. The area sizes of different parts of glomeruli (the detailed shown in Figure S2)^8^ were also roughly measured; SAMP8 mice had decreased relative capillary bulb area and increased renal vesicles area, indicating that with the kidney ageing progresses, the glomerular blood perfusion may reduce and leads to atrophy of glomeruli (Table S1).

### 2.2 The change of physiological indexes after dapagliflozin treatment

We intragastrically administrated the mice with dapagliflozin in different concentrations (detailed shown in Section 4) for 4 months. There were no significant differences for body weight, 24 h water intake, 24 h food intake and 24 h urine volume compared with SAMP8 model group after the whole treatment process. Blood glucose exhibited decreased trend in dapagliflozin treatment group on Month 2 and Month 3 time‐point compared with Model group, Dapa‐H group showed the significantly reduced blood glucose level. On Month 4, the blood glucose level in each group showed no difference. These results indicated that dapagliflozin administration could exert some hypoglycaemic effect in non‐diabetes surrounding, but with time prolonged, mice might be adapted to the effect and had no glucose level change. After dapagliflozin treatment, kidney weight increased, urine creatine and urine protein decreased compared with Model group, especially in Dapa‐H group (Table 1). Previous study had shown that the intervention of SGLT2i might decrease GFR during early treatment stage,^9^ so we measured tGFR (Transcutaneous glomerular filtration rate) values in Model group and Dapa‐H group after 1 month treatment. The results confirmed that SGLT2i could decrease kidney GFR at early treatment stage (Figure S1).

**TABLE 1:** Changes of metabolic indicators after dapagliflozin treatment.

	Model (*n* = 7)	Dapa‐L (*n* = 7)	Dapa‐M (*n* = 8)	Dapa‐H (*n* = 8)
Weight (g)				
Baseline	30.560 ± 1.637	29.930 ± 1.786	30.280 ± 1.331	31.140 ± 1.445
Month 1	31.990 ± 1.635	31.530 ± 2.317	32.110 ± 1.414	32.110 ± 1.414
Month 2	33.890 ± 1.068	33.390 ± 2.278	33.580 ± 2.361	33.440 ± 1.900
Month 3	36.390 ± 1.16	35.460 ± 2.898	36.160 ± 3.976	35.480 ± 1.988
Month 4	36.870 ± 1.400	36. 160 ± 3.483	37.940 ± 4.22	37.940 ± 4.22
Blood glucose (mmol/L)				
Month 1	7.600 ± 0.900	6.871 ± 0.991	7.125 ± 0.798	6.000 ± 1.156
Month 2	9.814 ± 1.624	7.329 ± 0.923	7.500 ± 1.206	6.963 ± 0.944*
Month 3	8.429 ± 0.980	9.386 ± 1.035	7.438 ± 1.341^‡^	7.075 ± 1.121^‡^
Month 4	7.757 ± 1.029	8.071 ± 0.869	8.125 ± 0.789	7.475 ± 1.120
Water intake (g/24 h)	2.329 ± 1.598	3.743 ± 2.638	2.643 ± 0.950	1.257 ± 1.883
Food intake (g/24 h)	3.571 ± 0.706	3.571 ± 0.706	3.663 ± 1.739	3.938 ± 1.176
Urine volume (mL/24 h)	0.886 ± 0.748	0.651 ± 1.106	0.795 ± 0.620	0.795 ± 0.620
Kidney weight (%)	0.038 ± 0.003	0.038 ± 0.003	0.041 ± 0.004	0.043 ± 0.004*
Urine creatine (μmol/L)	2673.000 ± 944.300	1912.000 ± 716.200	1729.000 ± 534.200	1293.000 ± 324.200*
Urine protein (μmol/L)	447.480 ± 157.390	349.330 ± 119.650	283.62 ± 86.927*	233.72 ± 29.577*

*Note*: Data presented are means ± SD. Model, SAMP8 control group; Dapa‐L, dapagliflozin low dosage group; Dapa‐M, dapagliflozin medium dosage group; Dapa‐H, dapagliflozin high dosage group.

*p < 0.05 vs. Normal, ^†^ *p* < 0.05 vs. Dapa‐L, ^‡^ *p* < 0.05 vs. Dapa‐M.

### 2.3 Effects of dapagliflozin on kidney histopathological senescence changes

**FIGURE 2:**
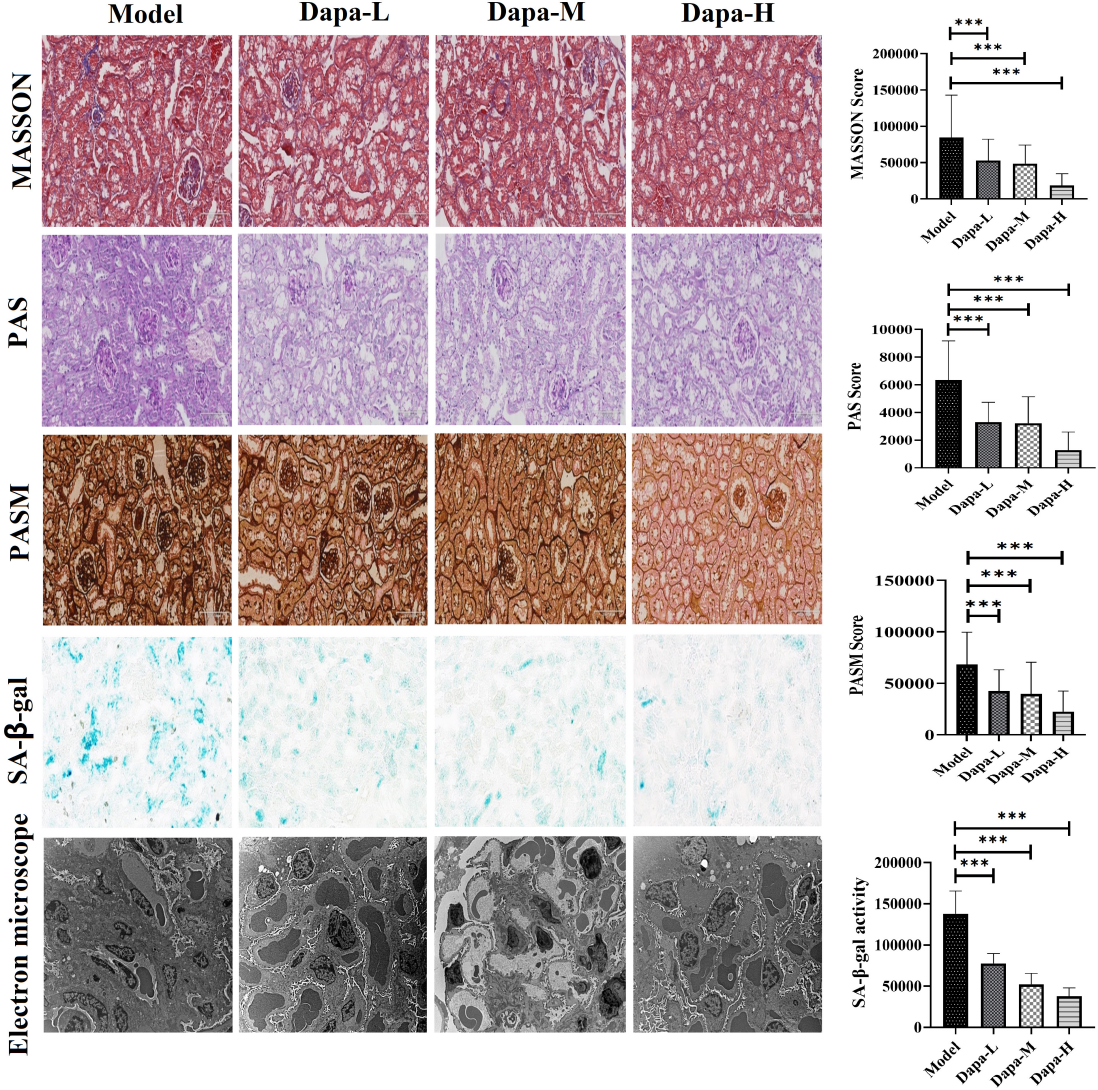
Effects of dapagliflozin on kidney histopathological senescence changes. PAS, periodic acid‐Schiff; PASM, periodic acid‐silver methenamine; SA‐β gal, senescence‐associated‐β‐galactosidase; Model, SAMP8 control group; Dapa‐L, dapagliflozin low dosage group; Dapa‐M, dapagliflozin medium dosage group; Dapa‐H, dapagliflozin high dosage group. *p < 0.05, **p <0.01, ***p <0.001.

Kidney samples of mice in different groups were resected, and staining related to renal fibrosis and glomerulosclerosis was conducted. Results showed dapagliflozin had beneficial effects on improving interstitial fibrosis and glomerulosclerosis, decreasing glomerular basement membrane, capillary basement membrane and tubulointerstitial collagen fibres deposition (Figure 2). Dapa‐H group exhibited clearer glomerular and tubular structures, neater arrangement of tubules (Figure 2). SA‐β‐gal activity staining also exhibited the lowest level in Dapa‐H group (Figure 2). Through detection by transmission electron microscope, dapagliflozin treatment improved glomerular mesangial hyperplasia (Figure 2). Treatment with dapagliflozin changed the area sizes of different parts of glomeruli, such as increasing relative capillary bulb area and decreasing renal vesicles area, indicating the glomerular atrophy can be improved by SGTL2i treatment (Table 2).

**TABLE 2:** Different area size of different parts of glomeruli in different groups.

	Glomerular area (μm^2^)	Capillary bulb area (μm^2^)	Relative capillary bulb area (μm^2^)	Relative renal vesicles area (μm^2^)
Model	6977 ± 1246	3196 ± 829.3	0.463 ± 0.112	0.537 ± 0.112
Dapa‐L	7207 ± 1286	3503 ± 879.3*	0.490 ± 0.078	0.510 ± 0.078
Dapa‐M	7062 ± 1434	3502 ± 864.7*	0.498 ± 0.069*	0.502 ± 0.069
Dapa‐H	6719 ± 1525	3496 ± 776.1*	0.520 ± 0.084*^†^	0.480 ± 0.084*^†^

*Note*: Data presented are means ± SD. Model, SAMP8 control group; Dapa‐L, dapagliflozin low dosage group; Dapa‐M, dapagliflozin medium dosage group; Dapa‐H, dapagliflozin high dosage group.

*p < 0.05 vs. Normal, ^†^ *p* < 0.05 vs. Dapa‐L, ^‡^ *p* < 0.05 vs. Dapa‐M.

### 2.4 Dapagliflozin decreased kidney and HK‐2 senescence markers

**FIGURE 3:**
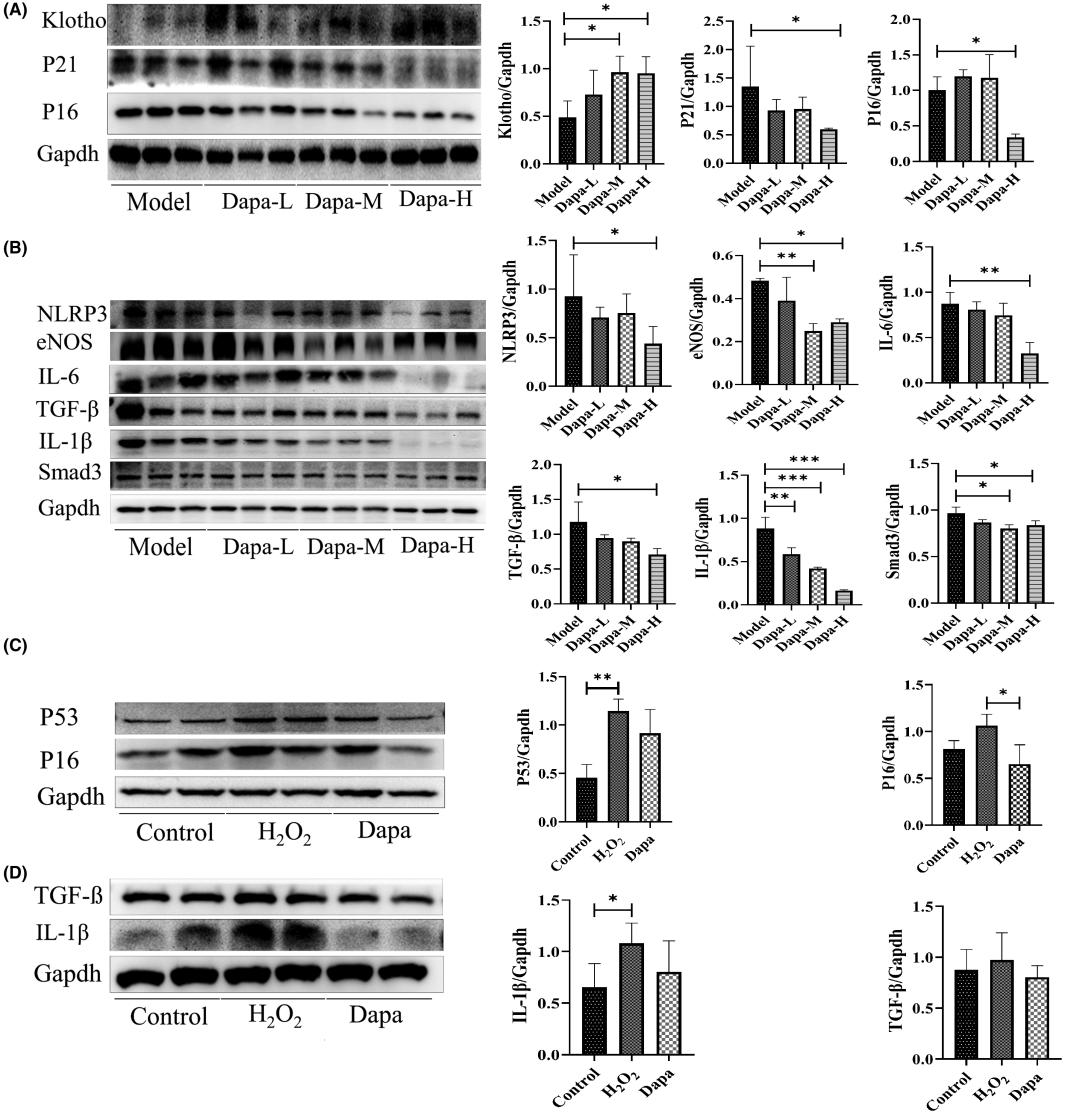
Dapagliflozin decreased kidney and HK‐2 senescence markers. (A) Kidney representative western blotting images and quantification results of Klotho, P21 and P16; (B) Kidney representative western blot images and quantification results of SASP (NLRP3, eNOS, IL‐6, TGF‐β, IL‐1β and Smad3); (C) HK‐2 cells representative western blotting images and quantification results of P53 and P16; (D) HK‐2 cells representative western blotting images and quantification results of SASP (TGF‐β and IL‐1β). Model, SAMP8 control group; Dapa‐L, dapagliflozin low dosage group; Dapa‐M, dapagliflozin medium dosage group; Dapa‐H, dapagliflozin high dosage group; *p < 0.05, **p < 0.01 and ***p < 0.001.

Western blot results revealed dapagliflozin decreased P21 and P16 protein expression, and increased klotho expression (Figure 3A). SASP (senescence‐associated secretory phenotype) protein expression, like NLRP3, eNOS, IL‐6, TGF‐β, IL‐1β and Smad3, also decreased after treatment (Figure 3B). HK‐2 cells were assaulted with H_2_O_2_ 200 μM for 6 h and then treated with dapagliflozin 20 μM for 48 h. The protein level of P53, P21, P16, TGF‐β and IL‐1 was measured; dapagliflozin decreased the increased level induced by H_2_O_2_ treatment (Figure 3C,D).

### 2.5 Screening the possible mechanism for the anti‐senescence role of SGLT2i by bioinformatics

**FIGURE 4:**
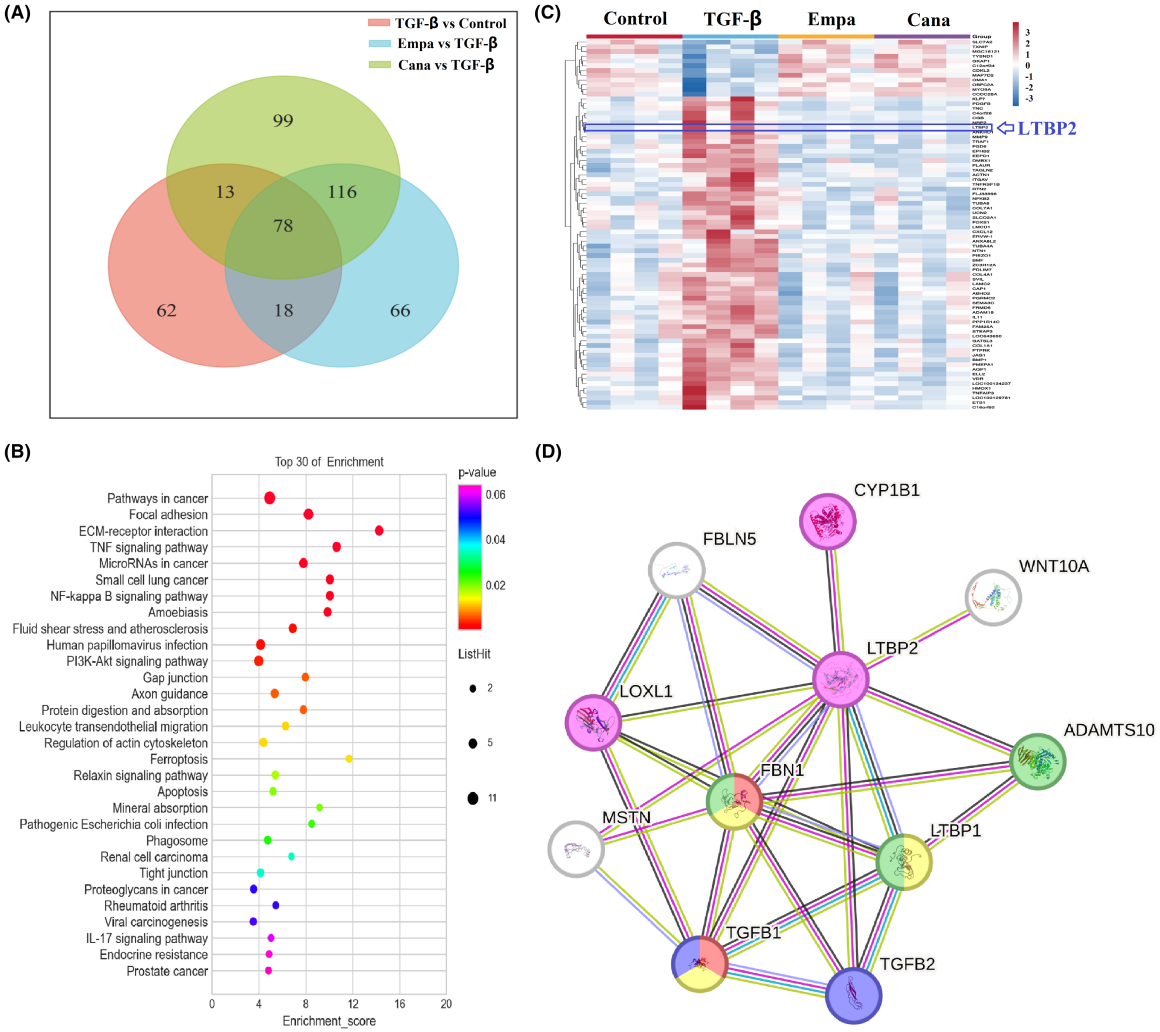
Exploring the possible mechanism for the role of SGLT2i anti‐senescence through bioinformatic analyses. (A) Venn diagram of different expressed genes between TGF‐β versus Control, Empa versus TGF‐β and Cana versus TGF‐β; (B) Bubble chart of KEGG pathways of 78 common different expressed genes enriched in; (C) Heatmap of the 78 common different expressed genes among the four groups (Control, TGF‐β, Empa and Cana); (D) PPI (protein–protein interaction) of LTBP2. Cana, canagliflozin; Empa, empagliflozin.

GSE118337 dataset^10^ from GEO (Gene Expression Omnibus) database was selected for further analyses. This dataset includes gene transcript data from four groups, namely unstimulated Control group, TGF‐β (10 ng/mL) group, Empagliflozin (500 nM) group and Canagliflozin (500 nM) group. We respectively got three parts of differential expressed genes between TGF‐β versus Control (171 genes), Empagliflozin versus TGF‐β (278 genes) and Canagliflozin versus TGF‐β (307 genes). Through intersection of the three parts of differential genes, we obtained the overlapped differential genes (78 genes) between the three parts (Figure 4A). KEGG pathway analyses showed the 78 differential genes mainly participated in pathways in cancer, focal adhesion and ECM–receptor interaction (Figure 4B). Expression of the 78 genes in different groups was displayed in heatmap (Figure 4C). Most of the genes were up‐regulated in TGF‐β treatment group and down‐regulated in empagliflozin and canagliflozin treatment groups. To identify which genes mostly be related to kidney senescence, we intersected these 78 genes with 2179 differential genes related to kidney senescence which data came from the kidney transcriptome sequencing comparation between 24 months and 3 months mice we did previously (data not shown). As a results, CCDC28A, TRAF1, TUBA8 and LTBP2 were considered to be the potential genes related to SGLT2i renal anti‐senescence function. Among them, LTBP2 showed the best potential ability that increasing with TGF‐β stimulation and decreasing after SGLT2i treatment. So LTBP2 was regarded as the possible target gene through which SGLT2i play its renal anti‐senescence role. Protein–Protein Interaction analyses from STRING showed LTBP2 might have many interactions with other proteins, like FBLN5,^11^ LOXL1,^12^ TGFβ1,^13^ TGFβ2,^14^ FBN1,^15^ MSTN, LTBP1, ADAMTS10, WNT10A and CYP1B1 (Figure 4D); most of these proteins play roles in extracellular matrix. So SGLT2i may play renal anti‐senescence role by LTBP2 through regulating extracellular matrix.

### 2.6 Validation of the renal anti‐senescence roles of LTBP2

**FIGURE 5:**
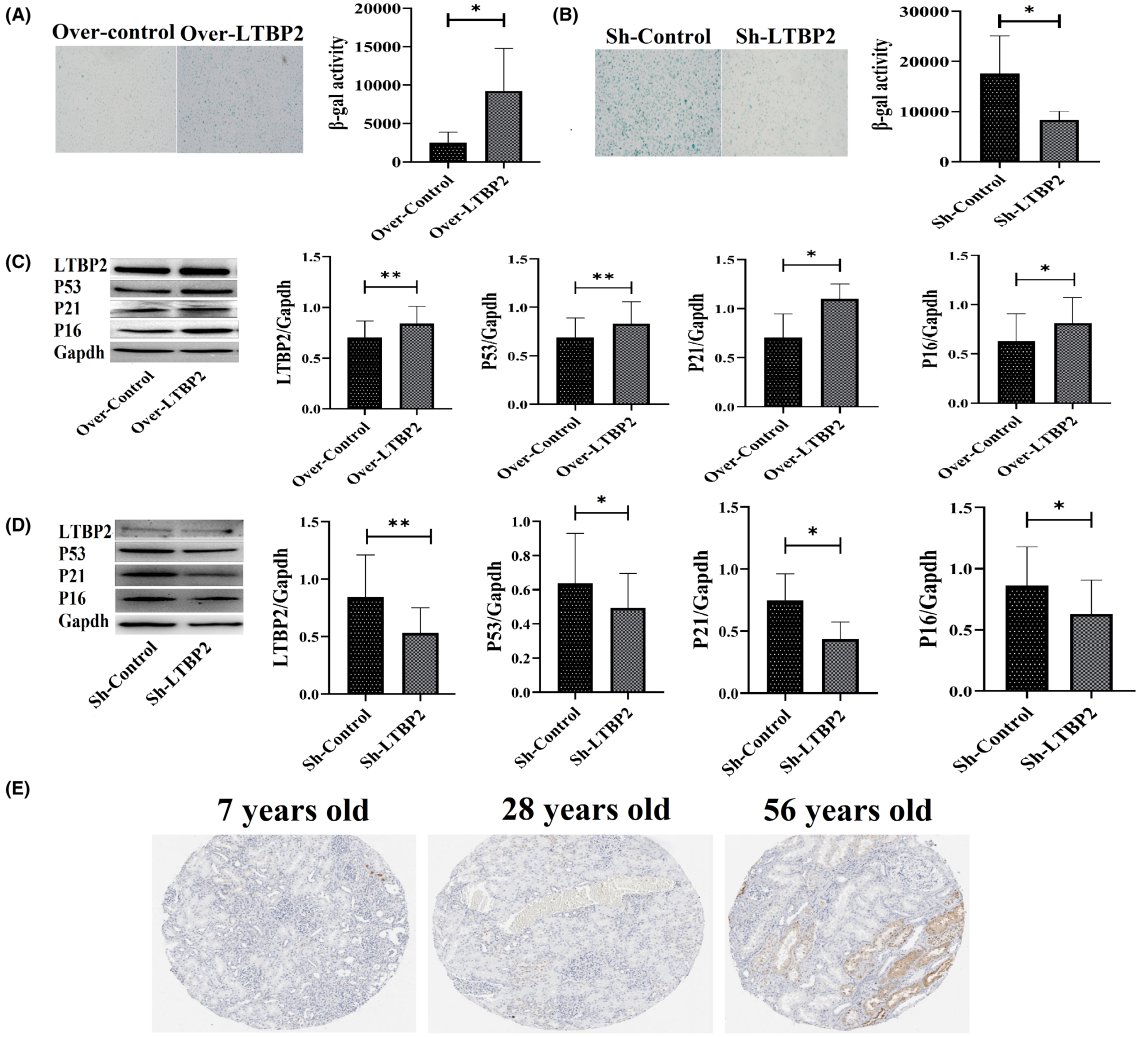
LTBP2 is associated with HK‐2 cells and kidney senescence. (A) Comparation of SA‐β gal staining between over‐expression control (Over‐control) and over‐expression LTBP2 (Over‐LTBP2) groups; (B) Comparation of SA‐β gal staining between knockdown control (Sh‐control) and knockdown LTBP2 (Sh‐LTBP2) groups; (C and D) HK‐2 cells representative western blotting images and quantification results of P53, P21 and P16, respectively, for over‐expression LTBP2 and knockdown LTBP2; (E) Immunohistochemical staining of different human kidney samples from different age (data got from database, detailed information showed in Section 4 parts). *p < 0.05, **p < 0.01 and ***p < 0.001.

To verify the renal anti‐senescence roles of LTBP2, LTBP2 over‐expressed and knockdown plasmid were separately constructed and transinfected into HK‐2 cells. SA‐β‐gal staining showed LTBP2 over‐expressed cells exhibited higher SA‐β‐gal activity (Figure 5A), and LTBP2 knockdown cells had lower SA‐β‐gal activity (Figure 5B). Western blotting results also indicated over‐expressing LTBP2 could increase the protein expression of P53, P21 and P16, vice versa knockdown LTBP2 cells expressed lower expression of these proteins (Figure 5C, D). Meanwhile, we explored the expression of LTBP2 in human kidney samples through The Human Protein Atlas database; three different ages kidney samples conducting LTBP2 immunohistochemical staining were displayed; the 56 year‐old kidney had the most prominent staining. All these results above revealed LTBP2 might participate into kidney senescence process.

### 2.7 Dapagliflozin reduced the expression of LTBP2 in vitro and in vivo renal senescence

**FIGURE 6:**
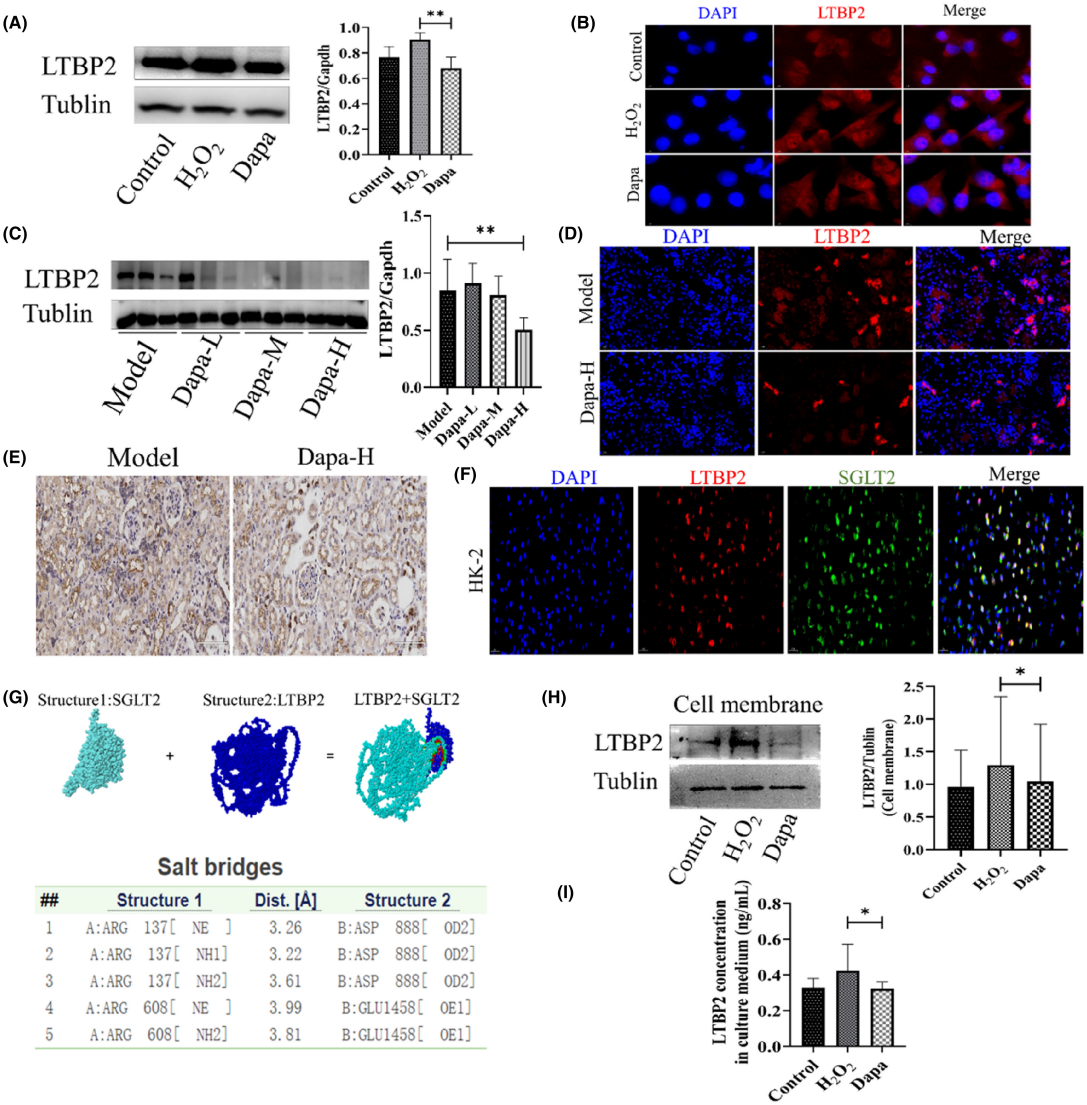
Dapagliflozin decrease LTBP2 expression induced by senescence both in vitro and in vivo. (A, C) HK‐2 and kidney representative western blotting images and quantification results for LTBP2; (B, D) HK‐2 and kidney immunostaining images for LTBP2; (E) Kidney immunohistochemical staining of LTBP2; (F) HK‐2 immunofluorescence colocalization for SGLT2 and LTBP2; (G) Protein–protein docking analyses between SGLT2 and LTBP2; (H) HK‐2 cells membrane western blotting for LTBP2 and quantification results; (I) Measurement of LTBP2 concentration in cell culture medium. Model, SAMP8 control group; Dapa‐L, dapagliflozin low dosage group; Dapa‐M, dapagliflozin medium dosage group; Dapa‐H, dapagliflozin high dosage group; *p < 0.05, **p < 0.01 and ***p < 0.001.

In vitro experiments, HK‐2 cells were assaulted with H_2_O_2_ to induce cell senescence, the expression of LTBP2 increased after H_2_O_2_ assault, dapagliflozin decreased LTBP2 expression (Figure 6A). Immunostaining results showed that LTBP2 expressed both in HK‐2 nucleus and cytoplasm, H_2_O_2_ assault increased LTBP2 expression in both two different areas of cells, while dapagliflozin decreased both of them (Figure 6B). In vivo experiments, dapagliflozin treatment reduced LTBP2 expression, especially in Dapa‐H group (Figure 6C). LTBP2 immunostaining and histochemistry staining of mice kidney exhibited the same results (Figure 6D,E). We concluded that LTBP2 might participated into the process of the renal anti‐senescence role of SGLT2i. For the reason that LTBP2 and SGLT2 both are membrane proteins, we hypothesized there were some relationships between LTBP2 and SGLT2. Immunofluorescence colocalization staining demonstrated there were colocalization between LTBP2 and SGLT2 (Figure 6F). Protein docking analyses of LTBP2 and SGLT2, using GRAMM‐X Protein–Protein Docking Web Server v.1.2.0 software,^16^ visualized the docking results through PDBePISA (Proteins, Interfaces, Structures and Assemblies) (https://www.ebi.ac.uk/pdbe/pisa/).^17^ Results showed that salt bridges existed between the two proteins. The interface area between the two proteins was 1881.4 A2; the docking free energy between the two proteins ΔG was −18.8 kcal/mol (Figure 6G). From the results above, we inferred that there might exist some weak connection between LTBP2 and SGLT2, SGLT2i could directly influence LTBP2 role through the weak connection. LTBP2 mainly play its role through secreting into extracellular matrix, so we separately measured LTBP2 expression level on cells membrane and in cell culture medium, compared the levels of LTBP2 expression in different treatment groups. LTBP2 cell membrane expression level increased after H_2_O_2_ assault and decreased after dapagliflozin treatment (Figure 6H), and the same was in cell culture medium (Figure 6I). All the results indicated SGLT2i might decreased the generation of intracellular LTBP2, and subsequently leading to the reduced LTBP2 level in extracellular matrix.

## 3 DISCUSSION

Ageing can predispose kidney state into acute and chronic kidney disease.^18^, ^19^, ^20^ Senescence in kidney can induce maladaptive repair and renal fibrosis. Exploring the appropriate strategy for delaying kidney ageing has great significance in improving the outcome of many different ageing‐related kidney diseases. Proximal tubules as the major component part of kidney, the senescence state of tubules could greatly affect kidney ageing progress. SGLT2i mainly focus on the sodium‐glucose cotransporter distributed on proximal tubular cells, through promoting the excretion of glucose to lower blood glucose and to exert beneficial effects.^21^ Although SGLT2i mainly focus on regulating physiologically glucose level, several researches have demonstrated that SGLT2i played beneficial roles transcending lowering blood glucose,^22^, ^23^, ^24^ the off‐target roles of SGLT2i were independent of lowering glucose.^18^, ^19^, ^25^ The renal anti‐senescence role of SGLT2i has been reported, most likely related to increasing the expression of klotho.^26^, ^27^ The evidence of renal anti‐senescence role of SGLT2i in natural kidney ageing model still be absent. In the current study, we aimed to testify whether SGLT2i could play anti‐senescence role under natural kidney ageing environment without diabetes and exploring the possible mechanism involved in the role. Several previous studies have demonstrated SAMP8 exhibited ageing‐related kidney features, such as increased urinary albumin ratio, glomerulosclerosis and tubulointerstitial fibrosis.^28^, ^29^ In our study, SAMP8 mice expressed higher level of renal ageing‐related markers and kidney ageing pathological structural changes compared with SAMR1 control mice, so SAMP8 strain mice were suitable for natural kidney senescence model. Treatment with dapagliflozin improved structural and functional features compared with Model group, such as increasing the loss of kidney weight, reducing urine creatine and proteinuria, ameliorating the extent of interstitial fibrosis and glomerulosclerosis, decreasing glomerular basement membrane, capillary basement membrane and tubulointerstitial collagen fibres deposition. SA‐β gal activity of kidney frozen sections was also alleviated by dapagliflozin treatment. The effects on kidney senescence makers, such as increasing klotho and decreasing P21, P16 and SASP (NLRP3, eNOS, IL‐6, TGF‐β, IL‐1β, Smad3) proteins expression strengthen the renal anti‐senescence role of dapagliflozin. In vitro experiment, dapagliflozin treatment on HK‐2 cells after insulting with H_2_O_2_ also displayed the role of alleviating cell senescence. The results above all demonstrated the anti‐renal senescence role of dapagliflozin. Our supplementary results showed dapagliflozin decreased the tGFR value compared with SAMP8 model group during the early treatment stage. However, the results of different area sizes changes of glomeruli and the images under transmission electron microscope at the end‐point of the experiment showed dapagliflozin treatment increased enrichment of capillary bulb and blood perfusion. Based on above results, we can deduce that although SGLT2i may decrease GFR at early treatment stage, in the long‐term treatment there may exist beneficial effects on glomerular perfusion. These were consistent with previous report that SGLT2i might induce an early, reversible reduction in GFR and can preserve GFR in the long‐term.^30^

Studies have showed that SGLT2i might play anti‐senescence role through sirt1, sirt3 and klotho activation,^27^, ^31^, ^32^, ^33^ to further identify the mechanism related to the role of SGLT2i delaying kidney senescence, we conducted some bioinformatics analyses through pubic databases. Through identifying the different expressed genes between different groups (Control, TGF‐β, Empagliflozin, Canagliflozin) and overlapping with senescence‐related transcript genes (the kidney different expressed genes between mice 3 month and mice 24 month) from our previous study. LTBP2 was selected as the candidate promising genes related to the renal anti‐senescence role of SGLT2is. The in vitro experiment results indicated regulating the expression of LTBP2 could affect the senescent state of cells. Immunohistochemical staining of LTBP2 in human kidney sample from public database also exhibited higher positive area in older one than younger one. These results above all proved that LTBP2 might be potential marker related to kidney senescence.

Subsequently experiments were conducted to verify whether SGLT2i could influence the expression of LTBP2 in vitro and in vivo. In vitro experiment, LTBP2 expression was elevated after H_2_O_2_ insultation and decreased after dapagliflozin treatment. In vivo experiment, LTBP2 was markedly down‐regulated by dapagliflozin intervention. Combining results from public database analyses and our laboratory experiments, we deduced that LTBP2 might be the potential target genes of SGLT2is to play the renal anti‐senescence role. SGLT2 and LTBP2 all present on cell membrane, co‐immunofluorescence staining of SGLT2 and LTBP2 in HK‐2 cells exhibited the overlapping expression between the two proteins. Protein docking between the two markers showed salt bridge formation; weak connections might exist between the two proteins. In vitro experiment, the increased LTBP2 after H_2_O_2_ stimulation was decreased by dapagliflozin. LTBP2 can secrete to extracellular matrix to prompt interstitial fibrosis; previous studies showed hyperglycaemic milieu is associated with LTBP2 secretion of cells.^34^, ^35^ In present study, we measured the concentration of LTBP2 in cell culture medium; dapagliflozin treatment decreased the secretion of LTBP2 into culture medium. These findings indicated that SGLT2i might play its role through inhibiting the intracellular expression of LTBP2, subsequently leading to the decreased secretion of LTBP2 into extracellular matrix and resulting in the alleviated renal fibrosis extent and attenuated renal senescence.

LTBP2 is not only an important component of ECM but also plays a pivotal role in TGF‐β activation.^7^ Previous study had suggested the supplement of exogenous LTBP2 to cultured fibroblasts resulted in TGF‐β activation in the medium.^36^ Several studies proved that LTBP2 had great association with tissue fibrosis, like heart and lung.^37^, ^38^ For the renal role of LTBP2, limited studies had been conducted. Apart from the unique ability of reabsorbing substances from the tubular filtrate, HK‐2 proximal tubule epithelial cells can also secret proteins into urine under normal conditions, and LTBP2 is one of the urinary secreted proteins.^39^ LTBP2 has been identified a new proximal tubule injury responsive gene.^34^ One research revealed the LTBP2 transcriptional changes in potential PT injury markers through single‐nucleus RNA sequencing (snRNA‐seq) in cisplatin‐induced nephrotoxicity models.^40^ The role of LTBP2 in other kidney disease models deserves further research. In the present study, we occasionally found that LTBP2 may participated in the renal anti‐senescence process of SGLT2i. Our results may shed a light on the role of LTBP2 in kidney disease in future.

In conclusion, the present research indicates that dapagliflozin can delay natural kidney senescence in non‐diabetes environment; the mechanism may be through regulating the role of LTBP2. The renal anti‐senescence role of SGLT2i and the role of LTBP2 in kidney ageing warrant further exploration.

## 4 MATERIALS AND METHODS

### 4.1 Animals and cells

Animal experiments were carried out in the Animal Experiment Center of Xi'an Jiaotong University, and the procedure complied with the Biomedical Ethics Committee of Medical Department of Xi'an Jiaotong University (2022‐1547). Male 3‐month‐old senescence‐accelerated mouse resistant 1 (SAMR1) and senescence‐accelerated mouse prone 8 (SAMP8) were purchased from the Medical Department of Peking University (Beijing, China). Mice were housed in a pathogen‐free environment with 22 ± 2°C temperature and 12 h light/dark cycle, and had free access to food and water. SAMP8 mice were divided into four groups, namely Model group (SAMP8 control group), Dapa‐L (dapagliflozin low dosage) group, Dapa‐M (dapagliflozin medium dosage) group and Dapa‐H (dapagliflozin high dosage) group. Model group mice were given sodium carboxymethyl cellulose containing DMSO at concentration same with other groups, Dapa‐L group was given dapagliflozin (Selleck, USA) at 1 mg/kg/d concentration; Dapa‐M group was given dapagliflozin at 2 mg/kg/d concentration; Dapa‐H group was given dapagliflozin at 4 mg/kg/d concentration; all drugs were given by intragastrically administration. The treatment time was 4 months. We measured the mice weight, blood glucose every month; blood glucose was got from cutting mice tails. The 24 h water intake, food intake and urine volume were measured through mouse metabolic cage by the end‐point of the experiment. Human kidney proximal tubular cells (HK‐2) were purchased from the Yuchicell (Shanghai) Biological Technology (Yuchi Biology, Shanghai, China) and cultured in DMEM/F12 (Biosciences, Shanghai XP Biomed, Shanghai, China). When the cells were 50%–60% confluent, the cells were insulted with 200 μM H_2_O_2_ for 6 h and then changed with cell culture medium containing dapagliflozin in 20 μM.

### 4.2 Urine chemistry

Creatinine and Urine Protein Quantitative Assay Kits (Nanjing Jiancheng, Nanjing, China) were used to measure urine protein and creatinine level; the procedures were conducted based on the product instructions.

### 4.3 Histopathology

Dissected kidney into small blocks and fixed in 4% formaldehyde, then embedded in paraffin and cut into 5 μm section. Separately evaluated the mesangial matrix expansion extent and interstitial fibrosis of kidney sections by Periodic acid‐Schiff (PAS) and Masson's trichrome staining. Each section was observed and photographed under microscope (Olympus CKX53, Japan). Positive staining area of each picture was quantified with Image‐Pro Plus 6.0 software (Media Cybernetics, USA).

### 4.4 Western blotting

Kidney and HK‐2 cells protein were extracted using RIPA buffer including protease inhibitor and PMSF (Heart, China). Samples protein concentration was measured by BCA Protein Quantitative Kit (Heart, China) and then adjusted to equal amounts for running western blot. Antibodies: anti‐p16 (1:1000; Abcam, England), anti‐p21 (1:1000; Abcam, England), anti‐p53 (1:1000; Abcam, England), anti‐Klotho (1:1000; Abcam, England), anti‐NLRP3 (1:1000; Cell Signaling Technology, USA), anti‐eNOS (1:1000; Cell Signaling Technology, USA), anti‐TGF‐β (1:1000; Abcam, England), anti‐IL‐6 (1:1000; Cell Signaling Technology, USA), anti‐IL‐1β (1:1000; Cell Signaling Technology, USA), anti‐Smad3 (1:1000; Proteintech, China), anti‐LTBP2 (1:1000; Santa cruz, USA), anti‐Tubulin (1:3000; Proteintech, China) and anti‐Gapdh (1:3000; Proteintech, China) were applied. Membranes were detected by chemiluminescence gel imaging system (FluorChem E; ProteinSimple, USA). Quantification of protein abundance was performed by Image J software 1.8.0.

### 4.5 Transcutaneous glomerular filtration (tGFR) rate measurement

Medibeacon company funded limited number machines for mice transcutaneous glomerular filtration (tGFR) rate measurement. We only selected three mice separately in Model group and Dapa‐H group for measuring tGFR after 1‐month dapagliflozin treatment. First, preparing FITC‐Sinistrin injection solution (0.07 mg FITC‐Sinistrin/1 g mouse weight) and removing suitable area of the mouse back hair, then fixing sensor machine on mice back and injecting FITC‐Sinistrin solution from tail vein, finally used Studio2 software for collecting data and analysing.

### 4.6 Bioinformatic analyses

From GEO (Gene Expression Omnibus) database, GSE118337 dataset^10^ was downloaded. This dataset included untreated control group, TGF‐β (10 ng/mL) stimulation group, empagliflozin (500 nM, 24 h) treatment group and canagliflozin (500 nM, 24 h) treatment group. R software was used to process the data. The differentially expressed genes (DEGs) between TGF‐β group versus control group, empagliflozin group versus TGF‐β group and canagliflozin group versus TGF‐β group were obtained through R software with ‘limma software’ package. The protein–protein interaction (PPI) network of the LTBP2 was constructed using STRING (version 12.0) (https://string‐db.org/).

### 4.7 Cell transfection

SiRNA targeting LTBP2 and non‐specific control were designed by Sangon Biotech. LTBP2 gene over‐expression vector plasmid and control empty plasmid were constructed and synthesized by WZ Biosciences. Transfection was carried out using Lipo8000 (Beyotime) according to the manufacturer's instructions.

### 4.8 Elisa

Human LTBP2 Elisa kit was bought from Cloud‐clone corp. HK‐2 cells were cultured in 6‐well plates and after treatment with different drugs; the cell culture medium was collected and centrifuged for 20 min under 1000 *g*; the supernatant was used to test LTBP2 concentration.

### 4.9 SA‐β‐gal assay

SA‐β‐gal staining of frozen sections of kidneys and cells was performed by using SA‐β‐gal staining kit (Cell Signaling Technology). The positive staining areas were evaluated by Image‐Pro Plus 6.0.

### 4.10 Immunostaining

Primary antibodies against LTBP2 (1:100, Santa Cruz Biotechnology, USA) and SGLT2 (1:100, Proteintech, China) were used.

### 4.11 Transmission electron microscope

Kidney ultrastructure was observed and photographed with transmission electron microscope (H‐7650, Hitachi, Japan).

### 4.12 Statistical analyses

Data are presented as the mean ± standard deviation (SD). The differences of data in more than two groups were evaluated using variance analyses. Data difference between two groups was analysed by two‐tailed Student's *t* test. Statistical analyses and plotting were performed using GraphPad Prism 8.0.2 (GraphPad Software, San Diego, CA). A value of *p* < 0.05 was considered to be statistically significant.

## AUTHOR CONTRIBUTIONS

**Lu Zeng:** Conceptualization (lead); data curation (lead); formal analysis (lead); investigation (lead); methodology (lead); writing – original draft (lead). **Jie Li:** Conceptualization (supporting); formal analysis (supporting); methodology (supporting). **Fanfan Gao:** Conceptualization (supporting); formal analysis (supporting); methodology (supporting). **Yangyang Song:** Data curation (supporting). **Limin Wei:** Methodology (supporting). **Ning Qu:** Methodology (supporting). **Shengnan Chen:** Methodology (supporting). **Xue Zhao:** Methodology (supporting). **Zitong Lei:** Methodology (supporting). **Wenya Cao:** Methodology (supporting). **Lei Chen:** Conceptualization (supporting); investigation (supporting); methodology (supporting). **Hongli Jiang:** Conceptualization (lead); funding acquisition (lead); investigation (lead); resources (lead); writing – review and editing (lead).

## CONFLICT OF INTEREST STATEMENT

The authors declare no conflict of interest.

## Supporting information


Appendix S1

## Data Availability

Data supporting the findings of the study are available from the corresponding author.
