# Supplementary material for: SGLT2i improves kidney senescence by down‐regulating the expression of LTBP2 in SAMP8 mice
Source: J Cell Mol Med. 2024 Mar 7;28(6):e18176. doi: 10.1111/jcmm.18176 (PMC10921069; doi:10.1111/jcmm.18176)
Supplement: Supplementary file 1 — Appendix S1 [file JCMM-28-e18176-s001.docx]

**SUPPORTING INFORMATION**


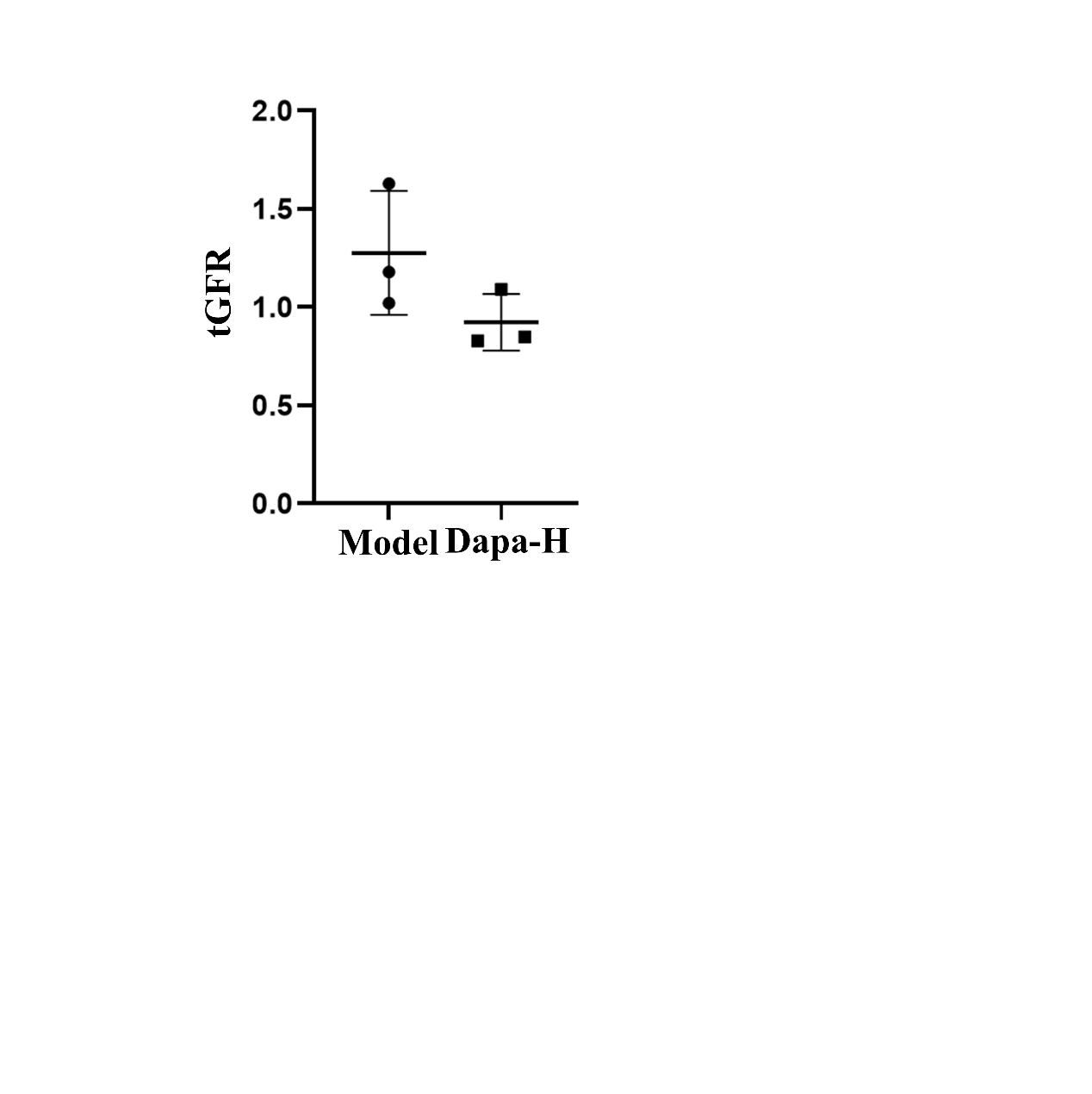


**Supplementary figure 1. The tGFR after 1 month of dapagliflozin treatment.**
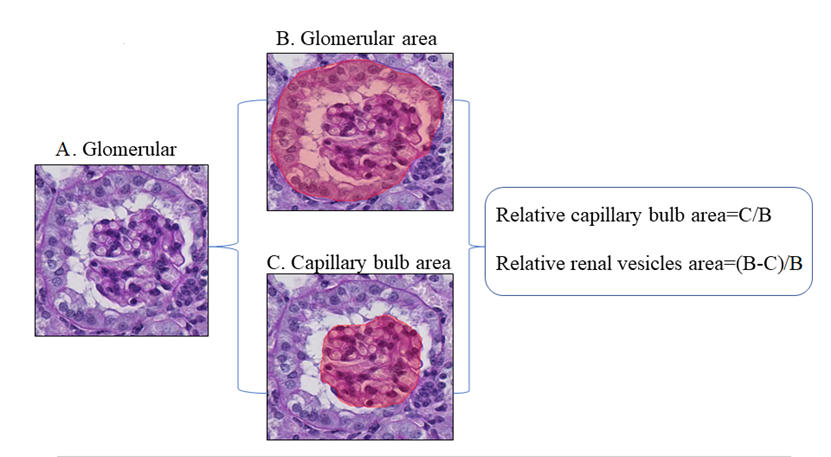


**Supplementary figure 2. The detailed illustration of different part area size of glomerular** (Zeng et al., 2023). A. intact glomerular; B. glomerular area (pink shadow area); C. capillary bulb area (pink shadow area).

**Supplementary table 1. The** **composition area size of different parts of glomeruli between SAMR1 and SAMP8.**

|  | Glomerular area（μm^2^） | Capillary bulb area（μm^2^） | Relative capillary bulb area（μm^2^） | Relative renal vesicles area（μm^2^） |
| --- | --- | --- | --- | --- |
| SAMR1 | 3514.50 | 6461.00 | 0.55 | 0.45 |
| SAMP8 | 3375.00 | 7260.00 | 0.49 | 0.51 |
| p value | 0.68 | 0.00 | 0.00 | 0.00 |
